# Supplementary material for: Resolving the data asynchronicity in high-speed atomic force microscopy measurement via the Kalman Smoother
Source: Sci Rep. 2020 Oct 27;10:18393. doi: 10.1038/s41598-020-75463-1 (PMC7592071; doi:10.1038/s41598-020-75463-1)
Supplement: Supplementary file 1 — Supplementary information 1. [file 41598_2020_75463_MOESM1_ESM.pdf]

Supplementary Figures for

Resolving the data asynchronicity in high-speed atomic force microscopy measurement via the Kalman Smoother

Shintaroh Kubo[1], Suguru Kato[1], Kazuyuki Nakamura[2], Noriyuki Kodera[3], and Shoji Takada[1]*

1) Department of Biophysics, Graduate School of Science, Kyoto University, Kyoto 606-8502, Japan 2) School of Interdisciplinary Mathematical Sciences, Meiji University, 4-21-1 Nakano, Nakano-ku, Tokyo 164-8525, Japan, and JST, PRESTO, 4-1-8 Honcho, Kawaguchi, Saitama, 332-0012, Japan, 3) Nano Life Science Institute, Kanazawa University, Kakuma-machi, Kanazawa 920-1192, Japan.

*Corresponding author: takada@biophys.kyoto-u.ac.jp (ST)

Short title: Resolving asynchronicity in HS-AFM

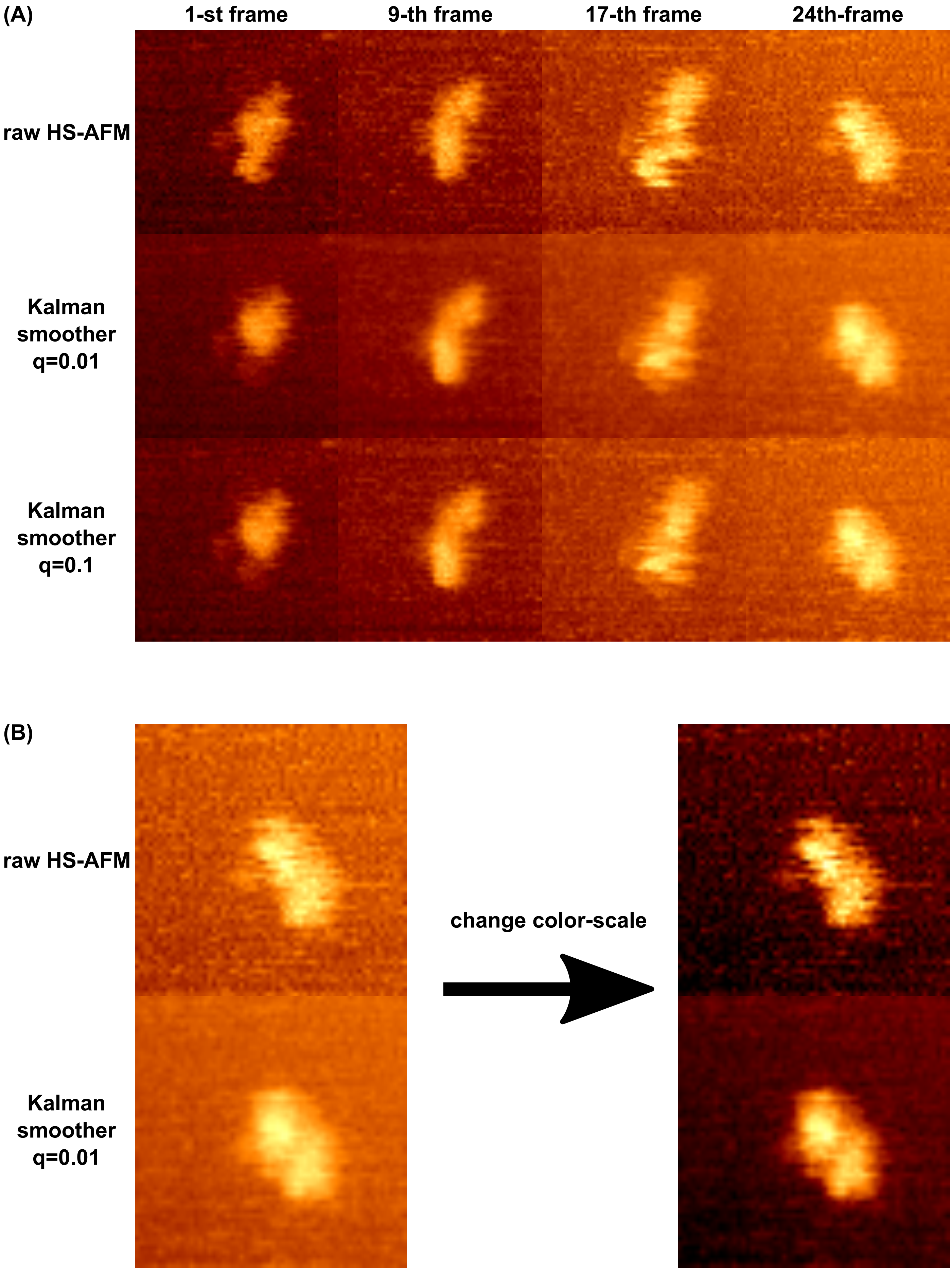

Figure S1. (A) Reproduction of Figure 10B images without contours. (B) A close-up view of the 24-th frame with a different color scheme.

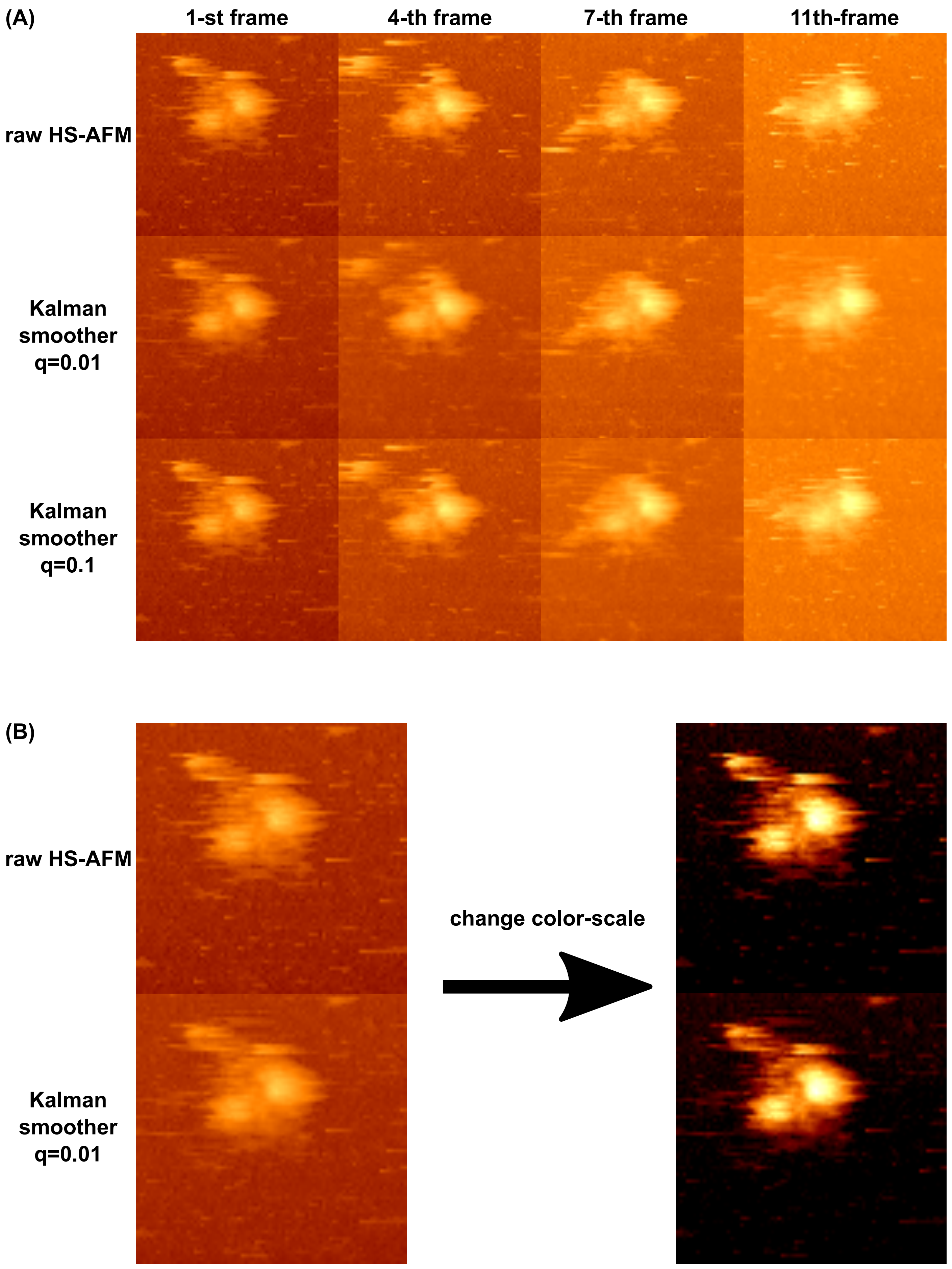

Figure S2. (A) Reproduction of Figure 11B images without contours. (B) A close-up view of the 1-st frame with a different color scheme.

Supplemental Figures and Movies

Figure S1. (A) Reproduction of Figure 10B images without contours. (B) A close-up view of the 24-th frame with a different color scheme.

Figure S2. (A) Reproduction of Figure 11B images without contours. (B) A close-up view of the 1-st frame with a different color scheme.

Movie S1. A ground-truth AFM-like movie of a diffusing cone.

Movie S2. A raw AFM-like movie of a diffusing cone.

Movie S3. A movie obtained by the Kalman filter method (q=0.1) for a diffusing cone.

Movie S4. A movie obtained by the Kalman smoother method (q=0.1) for a diffusing cone.

Movie S5. A ground-truth AFM-like movie of a dynein conformational change dynamics

Movie S6. A raw AFM-like movie of a dynein conformational change dynamics

Movie S7. A movie obtained by the Kalman smoother method (q=0.01) for a dynein conformational change dynamics

Movie S8. A HS-AFM of $FlhA_C$

Movie S9. A movie obtained by the Kalman smoother method (q=0.01) for $FlhA_C$

Movie S10. A HS-AFM of centralspindlin

Movie S11. A movie obtained by the Kalman smoother method (q=0.01) for centralspindlin
